# Comment on “microRNAs in the Same Clusters Evolve to Coordinately Regulate Functionally Related Genes”

**DOI:** 10.1093/molbev/msz120

**Published:** 2019-05-17

**Authors:** Antonio Marco

**Affiliations:** School of Biological Sciences, University of Essex, Colchester, United Kingdom

MicroRNAs are often clustered in the genome and it has been hypothesized that clustered microRNAs share common functions. However, statistical support for this hypothesis is lacking. Recently, Wang et al. (2016) stated that clustered microRNAs evolve to co-ordinately regulate common genes, targeting more common genes than expected by chance (*P* < 0.001; their fig. 3*D*). I explored their results in detail to identify potential clusters of interest. When their methodology is reproduced, clustered microRNAs had more common targets than expected by chance (*P* = 0.0350; Marco, 2018) but this was due to the presence of two clustered microRNAs with very similar sequences (and therefore similar targets). After removing this cluster the result was no longer significant (*P* = 0.2753). In general, the similarity between microRNAs is what determines the number of common targets, and not whether these microRNAs are clustered or not (Marco 2018).

After my reanalysis, Wang et al. acknowledged that the methodology used was different. Wang et al. (2016) wrote: “in the permutation analysis, we only randomly shuffled the locations of miRNAs.” However, Wang et al. (2018) reported instead that “[they] first shuffled the co-expressed seed: target pairing.” This point is critical as the reviewers of the original paper were unaware of the actual permutation method used, and this permutation method produces erroneously low *P*-values. As a simple example, let’s assume that we have three microRNAs, two clustered and one nonclustered, all targeting a common gene *a*. Additionally, each microRNA targets two other genes. Gene *a* is a promiscuous target that is targeted by all microRNAs in the network. It is evident that the two clustered microRNAs do not have more common targets than expected by chance. However, if we shuffle the interactions instead of the loci, it can be shown that the permutation test will find a significant enrichment of common targets for clustered microRNAs with a *P*-value of 0.000397. If instead of 2, each microRNA has three additional targets, the *P*-value will be 1.8 × 10^−6^ (see details in Marco 2018).

I also reconstructed 10,000 random microRNA data sets in which the “clusters” are formed by actually nonclustered microRNAs. If the test is unbiased, according to statistical theory the distribution of *P*-values for this data set must follow a uniform distribution. However, the distribution of *P*-values reported when the method by Wang et al. is used is extremely L-shaped (see fig. 3 in Marco 2018): indeed, the test detects that the majority of random cluster configurations has a significant enrichment in common targets. This permutation method has been used throughout in Wang et al. (2016, 2018) affecting most results reported. A full description of this analysis, the computing code used, as well as an extended discussion can be found in Marco (2018). In the associated preprint I also discuss evidence that the evolutionary dynamics of microRNA: gene interactions are mostly due to changes in target sites rather than at microRNA loci.

In conclusion, there is not statistical evidence that clustered microRNAs target more common genes than expected by chance, and there is no current evidence of widespread functional co-adaptation between clustered microRNAs.
